# Beyond the Cuff: State-of-the-Art on Cuffless Blood Pressure Monitoring

**DOI:** 10.3390/s26041243

**Published:** 2026-02-14

**Authors:** Yaheya Shafti, Steven Hughes, William Taylor, Muhammad A. Imran, David Owens, Shuja Ansari

**Affiliations:** James Watt School of Engineering, University of Glasgow, Glasgow G12 8QQ, UK; 3025970h@student.gla.ac.uk (S.H.); william.taylor@glasgow.ac.uk (W.T.); muhammad.imran@glasgow.ac.uk (M.A.I.); david.owens@glasgow.ac.uk (D.O.); shuja.ansari@glasgow.ac.uk (S.A.)

**Keywords:** acoustic, blood pressure, capacitive, cuffless, optical, radar, sensing

## Abstract

Blood pressure (BP) monitoring is crucial for identifying high BP (hypertension) and is an important aspect of patient care. However, traditional cuff-based methods for BP monitoring are unsuitable for continuous monitoring and can cause discomfort to patients. This survey critically examines the emerging field of cuffless BP monitoring, highlighting advances beyond traditional cuff-based methods. Technologies such as radar, optical, acoustic, and capacitive sensors offer the potential for continuous, non-invasive BP estimation, enabling applications in remote health monitoring and ambient clinical intelligence. We introduce a unifying taxonomy covering sensing modalities, physiological measurement principles, signal processing techniques, and translational challenges. Emphasis is placed on methods that eliminate subject-specific calibration, overcome motion artifacts, and satisfy international validation standards. The review also analyses Machine Learning (ML) and sensor fusion approaches that enhance predictive accuracy. Despite encouraging results, challenges remain in achieving clinically acceptable accuracy across diverse populations and real-world conditions. This work delineates the current landscape, benchmarks performance against gold standards, and identifies key future directions for scalable, explainable, and regulatory-compliant BP monitoring systems.

## 1. Introduction

Blood pressure (BP) is a vital physiological parameter and a key indicator of cardiovascular health. Accurate and continuous BP monitoring is essential for diagnosing and managing conditions such as hypertension, which affects more than a billion individuals globally [1,2] and contributes significantly to the burden of stroke, heart failure, and renal disease [3,4]. Elevated BP increases the workload on the heart and arteries, thereby raising the risk of cardiovascular complications and organ failure [5,6]. Clinical guidelines, such as those from the American Heart Association (AHA), define hypertension as Systolic BP (SBP) ≥ 130 mmHg or Diastolic BP (DBP) ≥ 80 mmHg [7], emphasizing the need for reliable measurement techniques.

Traditionally, BP has been measured using cuff-based methods. These include the auscultatory technique using mercury or aneroid sphygmomanometers and oscillometric devices. Mercury sphygmomanometers, once considered the gold standard [8,9], are now discouraged due to mercury toxicity. Oscillometric devices, which are currently recommended, offer automated and user-friendly BP measurement. However, all cuff-based methods are intermittent, can be uncomfortable, and may induce white-coat hypertension—an anxiety-related elevation in BP during clinical visits [10].

The true gold standard for BP measurement remains invasive intra-arterial monitoring, which provides continuous and highly accurate readings. Despite its precision, this method is impractical outside operating rooms or intensive care settings. These limitations have driven the development of *cuffless* BP monitoring techniques, which aim to provide continuous, unobtrusive, and potentially more representative BP readings in everyday environments [11].

Among cuffless methods, Photoplethysmography (PPG) has gained prominence. PPG measures blood volume changes in the microvascular bed of tissue using light absorption. During systole, increased blood volume leads to greater light absorption (signal trough), while diastole corresponds to reduced absorption (signal peak). These periodic changes in skin color and light absorption form the basis of PPG signals [12]. Remote PPG (rPPG), a cuffless variant, captures similar signals using cameras [13], enabling BP monitoring without physical contact—ideal for telemedicine, public health screening, and wearable technologies [14].

Another widely studied approach is Pulse Arrival Time(PAT), which measures the time delay between the electrical activity of the heart (via ECG) and the arrival of the pulse wave at a peripheral site (via PPG). It is important to distinguish PAT fromPulse Transit Time (PTT), which requires two PPG signals and excludes the Pre-Ejection Period (PEP) [15]. PAT and PTT are both influenced by arterial stiffness, which correlates with intra-arterial pressure, forming the physiological basis for BP estimation [16]. Additionally, Pulse Wave Analysis (PWA) is used to extract waveform features from PPG or other signals to estimate BP.

Radar-based technologies offer a promising cuffless approach to BP monitoring by detecting micro-movements of the chest wall and arteries induced by cardiac activity [17,18]. These subtle displacements can be analyzed to extract physiological features such as heart rate, PAT, and arterial motion. Through advanced signal processing and Artificial Intelligence (AI) techniques, these features can be used to estimate BP [19,20,21]. Unlike optical methods, radar systems are unaffected by skin tone or ambient lighting conditions, making them particularly suitable for calibration-free, continuous monitoring across diverse populations [22]. This robustness positions radar-based BP monitoring as a viable solution for clinical environments where inclusivity and reliability are paramount [23].

Despite increasing interest in cuffless BP monitoring, existing survey papers often lack comprehensive coverage of recent advances, particularly in remote sensing modalities and Machine Learning (ML)-based estimation techniques. Moreover, many reviews do not adequately address the clinical relevance, validation challenges, or the transition from cuff-based to cuffless methods. This review highlights the requirements of validation standard required for cuff-based and cuffless devices so that any future research can be clear on what requirements are needed for new devices to be clinically approved.

This survey aims to fill these gaps by achieving the following:

- Providing a clinically grounded overview of BP monitoring methods;
- Reviewing traditional and cuffless techniques, with emphasis on cuffless approaches;
- Clarifying key physiological principles and signal processing methods;
- Comparing existing surveys and identifying overlooked areas;
- Highlighting emerging trends, challenges, and future directions.

Section 2 provides a taxonomy of cuffless BP monitoring and an overview of the metrics and standards used to assess BP monitors. Section 3 discusses in detail the current research in each sensing approach for cuffless BP monitoring. Section 4 examines the different measurements that can be used to estimate BP. Section 5 details the signal processing techniques used in the current literature to extract the features needed to estimate BP. Section 6 discusses the major challenges in developing a clinically validated cuffless BP monitor and the approaches for overcoming these challenges.

### Design and Methods of Review

The aim of this review is to synthesize recent advances in cuffless BP monitoring technologies and identify gaps for future research. A systematic search was conducted across Google Scholar, MDPI, ScienceDirect, and IEEE Xplore databases. The following keywords and their variations were used: Cuffless BP monitoring; Non-invasive BP estimation; Contactless BP measurement; Continuous BP monitoring; Remote health monitoring BP; Calibration-free BP estimation; Radar-based BP monitoring; FMCW radar BP estimation; Ultra-wideband radar BP; Millimeter-wave radar BP; Optical BP monitoring; rPPG; Video-based BP estimation; Thermal imaging BP; Acoustic BP monitoring; Doppler ultrasound BP estimation; Ballistocardiography (BCG); Seismocardiography (SCG); Capacitive sensing; Electric field sensing; PTT BP estimation; PAT; Pulse Wave Velocity (PWV); Micro-Doppler radar; Hemodynamic response BP estimation; PPG signal derivatives for BP estimation; Time-domain BP features; Frequency-domain BP analysis; Wavelet transform BP estimation; Deep learning (DL) BP estimation; CNN LSTM BP prediction; ML BP estimation; Multimodal sensor fusion BP; Motion artifact BP monitoring; Clinical validation cuffless BP; AAMI BP validation standards [24]; BHS BP validation protocol [25]; ISO 81060-2 cuffless BP standards [26]. Paper selection was based primarily on relevance to cuffless BP monitoring and secondarily on publication date, with preference given to recent peer-reviewed studies.

## 2. Taxonomy of Cuffless BP Measurement

**Figure 1:**
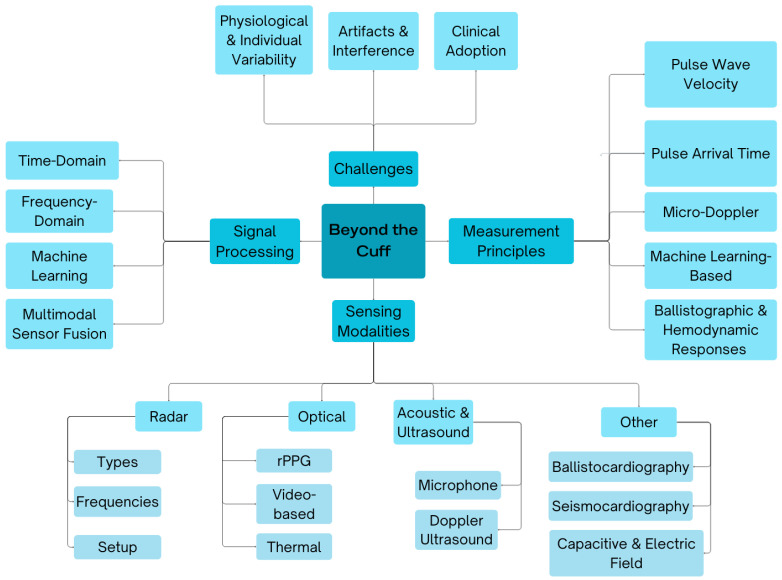
Taxonomy of cuffless BP estimation.

Figure 1 presents a comprehensive taxonomy of the current landscape in cuffless BP monitoring, as explored in this review. We structure the field into four principal domains: Sensing Modalities, Measurement Principles, Signal Processing, and Challenges.

Under Sensing Modalities, we categorize the diverse sensor technologies enabling cuffless BP estimation into Radar (including radar types, operating frequencies, and experimental setups), Optical (spanning rPPG, video-based, and thermal imaging), Acoustic & Ultrasound (microphone and Doppler ultrasound), and other techniques (BCG, SCG, and capacitive or electric field-based methods). Measurement Principles outline the underlying physiological and computational mechanisms for BP estimation, including PTT and PWV, micro-Doppler-based methods, ML-driven approaches, and those leveraging ballistographic or hemodynamic responses. Signal Processing approaches are divided into time-domain, frequency-domain, and ML-based methods, with an increasing emphasis on multimodal sensor fusion to improve accuracy and robustness. Finally, we delineate the challenges that hinder clinical translation, including physiological and individual variability, motion artifacts and environmental interference, and the broader issues surrounding regulatory and clinical adoption.

This taxonomy serves as a unified framework for understanding the interdisciplinary efforts converging in the emerging domain of cuffless BP monitoring, while also guiding future research directions.

The clinical validation of BP devices requires rigorous testing protocols to ensure measurement accuracy, reliability, and reproducibility across diverse populations and physiological conditions. International standards, such as those established by the British Hypertension Society (BHS) [25], Association for the Advancement of Medical Instrumentation (AAMI) [24], International Organization for Standardization (ISO) (e.g., ISO 81060-2:2018 [26]), and the European Society of Hypertension (ESH) [27], define strict criteria for participant selection, measurement procedures, and statistical performance thresholds. These protocols typically require validation across a broad range of BP values, age groups, and arm circumferences, with independent comparisons against a reference device (gold standard). Furthermore, repeatability under varying conditions, such as motion, temperature changes, and skin tone variability, is increasingly emphasized, particularly for novel cuffless methods. Ethical approval and appropriate clinical supervision are also essential to ensure data integrity and participant safety. The following section explores how cuffless BP systems have been benchmarked against traditional gold-standard devices, highlighting both achievements and existing performance gaps.

### 2.1. Benchmarking Against Gold-Standard BP Devices

To assess the performance and reliability of the BP monitoring system, a set of evaluation and validation metrics must be employed. The metrics are selected with the intention of providing insights into both the accuracy of the signal processing model and the clinical relevance of its predictions. The metrics used are as follows.

#### 2.1.1. Mean Absolute Error and Standard Deviation

The Mean Absolute Error (MAE) measures the average error magnitude between predicted and actual BP values received from the gold-standard BP device. It is expressed in mmHg and is mathematically calculated as

(1) $$\mathrm{MAE}=\frac{1}{n}\sum_{i=1}^{n}\left|p_{i}-a_{i}\right|,$$

where $p_{i}$ is the predicted BP value, $a_{i}$ is the actual gold-standard BP value, and *n* is the number of instances per subject. A low MAE magnitude indicates higher accuracy [28].

The Standard Deviation (SD) is used to measure variability in the prediction errors and is expressed in mmHg; it is mathematically calculated as

(2) $$SD=\sqrt{\frac{1}{n}\sum_{i=1}^{n}{\left(p_{i}-a_{i}-\bar{e}\right)}^{2}},$$

where $p_{i}$ is the predicted BP value, $a_{i}$ is the actual gold-standard BP value, $\bar{e}$ is the mean error, and *n* is the number of instances per subject. A low value of SD indicates that the model’s predictions are closer to the actual ground-truth values.

In the literature, the accuracy of the system is displayed as MAE±SD [28]. Hence, finding these parameters is useful, particularly while comparing results with other systems.

#### 2.1.2. Root Mean Squared Error

The Root Mean Squared Error (RMSE) emphasizes larger errors as the differences are squared and are useful in cases of models making significant mis-predictions. It can be mathematically represented as

(3) $$\mathrm{RMSE}=\sqrt{\frac{1}{n}\sum_{i=1}^{n}{\left|p_{i}-a_{i}\right|}^{2}},$$

where $p_{i}$ is the predicted BP value, $a_{i}$ is the actual gold-standard BP value, and *n* is the number of instances per subject. This metric is useful and important in clinical settings where precision is a top priority [28].

#### 2.1.3. Correlation Coefficient (R or Pearson’s r)

This metric evaluates the linear correlation between actual and predicted values. The metric has been used to evaluate the linear relationship of BP with PAT in the literature [29]. The correlation coefficient or Pearson’s r can be mathematically represented as

(4) $$r=\frac{\sum_{i=1}^{n}\left(a_{i}-\bar{a}\right)\left(p_{i}-\bar{p}\right)}{\sqrt{\sum_{i=1}^{n}{\left(a_{i}-\bar{a}\right)}^{2}}\sqrt{\sum_{i=1}^{n}{\left(p_{i}-\bar{p}\right)}^{2}}},$$

where $p_{i}$ is the predicted BP value, $a_{i}$ is the actual gold-standard BP value, $\bar{a}$ is the mean of the actual values, $\bar{p}$ is the mean of the predicted values, and *n* is the number of instances per subject. The values for this correlation coefficient range from −1 to 1, with values closer to 1 indicating a strong positive correlation.

#### 2.1.4. Coefficient of Determination ($R^{2}$)

Beyond simple linear correlation between actual and predicted values, the coefficient of determination ($R^{2}$) expresses the proportion of variation in the actual values that is captured by the predictive model. Therefore, the coefficient of determination expresses the model’s ability to cope with variation in the test population, not the model’s overall accuracy [30]. The coefficient of determination can be mathematically represented as

(5) $$R^{2}=1-\frac{\sum_{i=1}^{n}{\left|p_{i}-a_{i}\right|}^{2}}{\sum_{i=1}^{n}{\left|\bar{a}-a_{i}\right|}^{2}}$$

#### 2.1.5. Bland–Altman Analysis

This technique assesses the clinical agreement between predicted and reference BP values against their mean. The Limits of Agreement (LoA) are defined as

(6) $$\mathrm{LoA}=\bar{d}\pm 1.96\cdot \mathrm{SD},$$

where $\bar{d}$ is the mean difference and SD is the SD. This method helps top identify any systematic bias; it assesses whether differences between methods are clinically acceptable and whether more refinement is needed [31].

Bland–Altman plots are often accompanied by scatter plots of predicted BP values vs. ground truth. These plots typically display a fitted regression line alongside a plot of the ideal line (y=x), as seen in [32]. This allows a visual assessment of any systematic calibration bias, range compression, or range expansion present in the results.

#### 2.1.6. Clinical Standards Comparison

The BHS and the AAMI standards have previously been used to validate cuff-based BP estimation systems against the gold-standard sphygmomanometer. ISO 81060-2:2018 is now the universal standard for cuff-based devices, replacing older AAMI/BHS protocols. For cuffless devices, the ESH is the relevant standard [27]. It includes criteria for different positions, post-exercise measurements, and calibration. Below is a description of each of the standards.

##### AAMI

The AAMI guidelines for assessing and grading the accuracy of cuff-based devices are based on mean difference and SD (mean±SD). Table 1 provides detailed information [24].

The main criteria for a device to fulfill the AAMI criteria are for it to have a mean difference < 5 mmHg and an SD < 8 mmHg [33]. Validation must be against a minimum of 85 participants [34].

##### BHS

The BHS guidelines for assessing the accuracy of cuff-based devices require a different set of calculations. Firstly, a metric is used that compares the absolute difference of the test device with the gold-standard sphygmomanometer and categorizes its accuracy. The categorization can be seen in Table 2 [35].

The BHS criteria for grading a cuff-based device can be seen in Table 3 [36]. Grades A to D are given to the device based on the percentage of readings falling below 5, 10, and 15 mmHg of the gold-standard sphygmomanometer.

For a BHS grade to be awarded, all three percentages (≤5 mmHg, ≤10 mmHg, ≤15 mmHg) corresponding to the grade must be greater than or equal to the values against them. The BHS standard also requires a minimum number of 85 participants for validation [34].

##### ISO 81060-2:2018

The ISO 81060-2:2018 standard has since replaced the older AAMI and BHS standards for cuff-based devices. This was implemented to globalize an industry standard for validating BP devices. The ISO 81060-2:2018 states that there must be a minimum of 85 participants for validation with 3 readings taken per participant and at least 30% of participants must be each male and female genders. The ISO 81060-2:2018 standard has a pass and fail criterion, with a pass criterion of less than or equal to ±5 mmHg and SD values of ≤8 mmHg [37].

##### ESH

The ESH standard is a protocol specifically for the validation of cuffless BP monitoring devices. The ESH is similar to the ISO 81060-2:2018 cuff-based standard but with added requirements for cuffless devices [27]. These added requirements include the following:

- Validation with baseline measurements, change in position, post-exercise, and post-treatment;
- Validation of calibration (initial calibration, stability of calibration, and consistent results of devices claiming no calibration requirements);
- Referenced against validated cuff-based systems or gold standard;
- Full disclosure of device type, sensing modality, and calibration procedure

##### Additional Cuffless Standards

Additionally there is an IEEE standard 1708-2014 and 1708a-2019 and ISO standard 81060-3:2022 protocols for cuffless BP monitoring devices. These standards also include additional requirements with the nature of cuffless monitoring, such as the calibration of devices. The pass requirements for IEEE standard 1708-2014 and 1708a-2019 is ≤7 mmHg (mean absolute difference) and for ISO standard 81060-3:2022 is ≤6 ± 10 mmHg (mean ± SD of difference) [27].

## 3. Sensing Modalities for Cuffless BP Monitoring

### 3.1. Radar-Based Methods

One of the most investigated sensing modalities for BP detection is radar, due to the precise detection of cardiac activity it provides. In addition, in contrast to a camera, there are no concerns regarding privacy, skin tone, or lighting. In principle, the radar transmits and receives reflected waves, detecting the displacement of the target, allowing a completely off-body and cuffless nature.

Various radar types and frequency bands are used for BP estimation. The types used are typically Continuous Wave (CW), Frequency-Modulated Continuous Wave (FMCW), and Ultra-Wide Band (UWB) radars. CW radars transmit and receive a continuous signal of constant frequency. They are used for their low cost and simplicity in design. However, they can be limited to short distances as they do not have range resolution and cannot eliminate interference from foreign reflections. The work conducted in both [38,39] successfully used 24 GHz CW radars, achieving results of 7.20 ± 6.73 mmHg and 6.30 ± 3.85 mmHg and of 4.84 ± 7.51 mmHg and 3.82 ± 5.81 mmHg (MAE ± SD for SBP and DBP), respectively. This proves them capable of estimating BP, especially through the power of strong signal processing and ML algorithms. However, it is possible that, in a real-time system, FMCW radars would prove to be better. FMCW radars provide the ability of range resolution and can typically achieve a better signal-to-noise ratio. Especially in a real-time system, their ability to limit detection within a specific range could prove useful for a better signal-to-noise ratio. FMCW radars do this as they transmit chirps, signals of sweeping frequency, allowing the calculation of both range and motion based on the frequency of the received signal. However, due to this advancement, they are more expensive than CW radars and more complex in design. Research in [40,41] achieved strong accuracy with FMCW radars (77 and 60 GHz respectively), showing results of 3.33 mmHg and 3.14 mmHg (RMSE) and 5.54 ± 4.79 mmHg and 4.18 ± 3.34 mmHg (MAE ± SD), respectively. Both studies took advantage of the range resolution by conducting Fast Fourier Transform (FFT) to create range-time maps and focus on the target signals. On the other hand, UWB radars can also achieve range resolution by transmitting pulses with a broad bandwidth. Studies using UWB radars argue that they display fewer harmonics, robustness towards interference, and a better SNR. Results of 6.5 ± 6.1 mmHg and 4.7 ± 4.9 mmHg (MAE ± SD) were achieved using a UWB radar in [36]. Other studies either extract PTT or the pulse wave, without any BP estimation. Looking at results with other radar types, they are accurate and comparable with [36]’s findings. Hence, the complexity and cost of a UWB radar may not be worth its use, especially when FMCW radars can also provide range resolution.

Frequencies range from 24 GHz to 300 GHz. Higher frequencies can detect smaller changes in displacement, potentially providing better resolution. However, lower frequencies provide lower power consumption and better penetration through clothes. In addition, the hardware is simpler, which can better serve commercialization efforts. A wide range of frequencies have achieved accurate results. A 300 GHz CW radar was used in [42], while ref. [39] used a 24 GHz one, both achieving accurate results.

There are various ways in which the radar can be set up for BP estimation. Typically, the target is the chest, wrist, neck, or a combination of these. All of these setups have shown strong results. The work in [40] recorded the chest and wrist to calculate PTT as a feature, ref. [43] recorded just the neck to extract the pulse wave for use in a DL algorithm, ref. [38] recorded just the chest and extracted features like systolic and diastolic time for ML, and ref. [21] recorded just the wrist to find the PTT. The setup is most important when considering the applicability and practicality of the potential device developed. Hence, most recent studies are either solely of the wrist with the arm rested, or of the chest with the subject sitting or lying. Chest, wrist, or neck recordings are all have a risk of motion artifacts affecting the readings. When attempting to use radar to perform cuffless BP monitoring, it is important that measurements are taken when users remain still and not during everyday activities. In addition, using just one radar will be advantageous as there are fewer variables and the system is simpler. Recordings typically range from 10 to 30 s if a “one shot” system is used [38,43]. Some studies instead use long continuous recordings of subjects, for example, ref. [44] used 10-min recordings of the subject’s chest while lying in bed. Measurement distances typically range anywhere from a few centimeters to 1 m [21,38,45].

### 3.2. Optical-Based Methods

**Figure 2:**
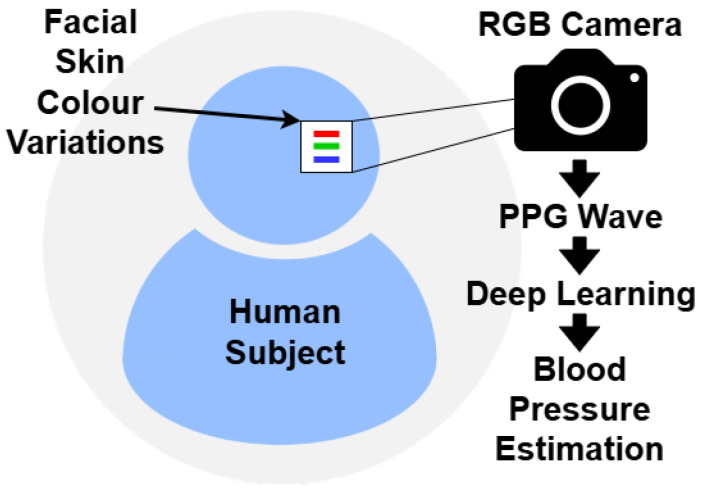
rPPG.

**Figure 3:**
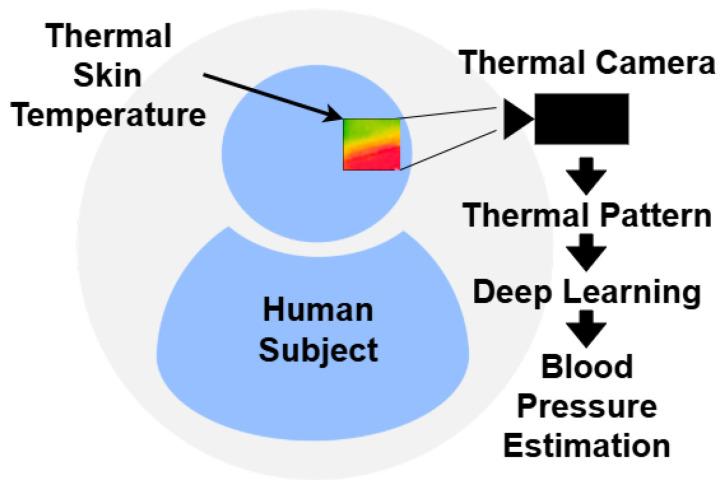
Thermal imaging techniques.

Optical-based techniques for physiological monitoring exploit the interaction of light with biological tissues to infer cardiovascular parameters. These methods have gained increasing attention for their potential in cuffless and continuous BP estimation, particularly in wearable and remote health monitoring applications. By analyzing subtle changes in light absorption, reflection, or emission, often through imaging modalities such as Red, Green, and Blue (RGB) cameras or thermal sensors, optical methods can capture hemodynamic signals indicative of BP. Unlike traditional cuff-based systems, optical approaches offer enhanced user comfort and long-term usability, thereby enabling scalable solutions for telehealth, ambient intelligence, and personalized medicine. Figure 2 and Figure 3 shows the common optical-based methods of rPPG and thermal-based methods.

#### 3.2.1. Remote Photoplethysmography

rPPG is a cuffless method that estimates cardiovascular parameters by analyzing facial skin color variations due to blood volume changes using RGB cameras. These changes are captured using consumer-grade RGB cameras. Recent methods have incorporated DL to map rPPG signals to BP values with improved generalization. Video-based methods use multiple RGB frames to extract physiological parameters such as PTT by analyzing the time difference between pulse waves detected at various facial regions. Single-image methods infer BP from static facial cues (e.g., skin tone, vascular texture) without temporal signals, making them lightweight and suitable for quick screening. However, they rely on indirect correlates and are less sensitive to short-term BP changes. Video-based approaches leverage rPPG or PTT to capture physiological dynamics, improving accuracy and responsiveness but requiring stable tracking, higher frame rates, and greater computational resources. In practice, single-image models favor convenience, while video-based methods offer stronger physiological grounding for continuous monitoring. Both face challenges from motion, illumination, and camera variability.

#### 3.2.2. Thermal Imaging Techniques

Thermal imaging leverages infrared sensors to monitor skin temperature variations, which are affected by underlying blood flow. ML models have been employed to correlate thermal patterns with resting BP [46]. While promising, these methods are sensitive to environmental factors and skin emissivity variations.

### 3.3. Acoustic- and Ultrasound-Based Methods

**Figure 4:**
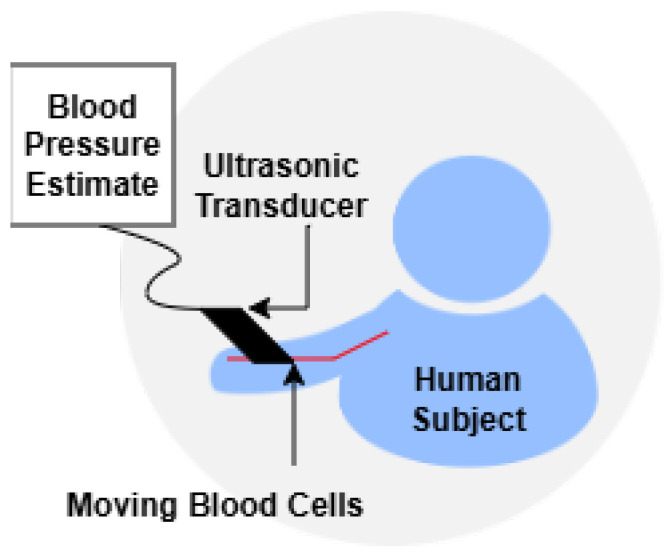
Doppler ultrasound BP estimation.

**Figure 5:**
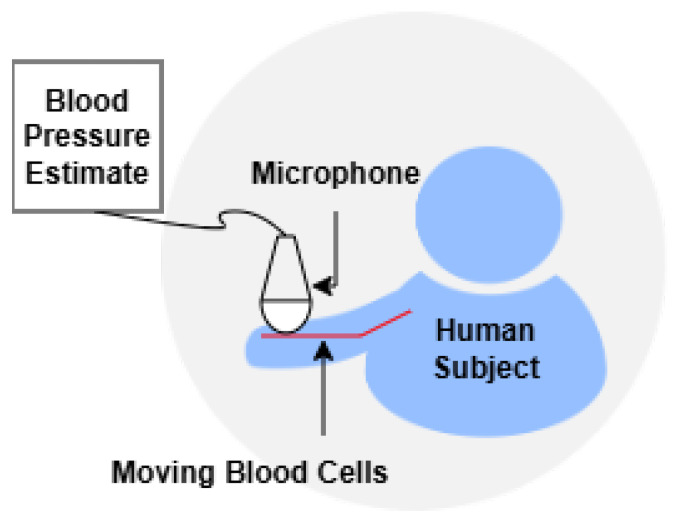
Acoustic-based BP measurement using microphones.

Acoustic- and ultrasound-based methods provide an alternative, non-invasive pathway for BP monitoring by capturing physiological signals through sound propagation. These techniques encompass the use of Doppler ultrasound to measure blood flow velocities and the application of microphones to record cardiovascular sounds, such as heartbeats and arterial pulse waves. The underlying principle relies on the acoustic characteristics of blood flow and vessel wall movement, which are modulated by BP variations. These methods are particularly valuable in clinical and ambulatory settings, offering real-time feedback and high temporal resolution. Recent advancements in sensor miniaturization and ML have further enhanced their feasibility for cuffless, continuous monitoring applications. Figure 4 and Figure 5 shows the Doppler ultrasound measures and acoustic-based BP measurements using microphone methods.

#### 3.3.1. Doppler Ultrasound BP Estimation

Doppler ultrasound measures blood flow velocities using the Doppler shift principle. It provides non-invasive estimates of SBP and DBP by analyzing the reflected signal from moving blood cells [47]. This method is especially useful in clinical and continuous monitoring contexts, though DBP accuracy is comparatively lower. Doppler ultrasound BP estimation requires contact or near contact on the skin and requires coupling gel. A trained operator is needed to operate ultrasound technology, as the sensors are highly sensitive to environmental noise and placement. This results in limitations in the practicality of ultrasound BP estimation.

#### 3.3.2. Acoustic-Based BP Measurement Using Microphones

This technique involves capturing cardiovascular sounds via microphones, including in-ear or wrist-mounted configurations. These sounds are then processed to estimate BP in real-time [48]. Similar to Doppler ultrasound BP estimation, acoustic-based BP measurements have limitations on practicality and accuracy due to ambient noise and sensor positioning and also require trained operators to ensure that the microphones can obtain the right information to accurately estimate BP in subjects. Nevertheless, acoustic-based BP measurements using microphones can provide greater comfort and portability than ultrasound technology.

### 3.4. Other Emerging Technologies

In addition to radar, optical, and acoustic modalities, several emerging technologies have shown promise in the domain of non-invasive and cuffless BP monitoring. These methods often rely on mechanical, inertial, or electrostatic interactions to capture cardiovascular dynamics. Notably, approaches such as BCG, SCG, and capacitive sensing provide valuable insights into cardiac mechanics and hemodynamic responses without the need for direct arterial access. These techniques are increasingly being integrated into wearable systems and smart environments, benefiting from advances in low-noise sensors and ML algorithms. However, BCG, SCG, and capacitive sensing are confined to controlled environments as motion artifacts and posture affect both the practicality and accuracy of these technologies. For use in real-world scenarios, calibration and custom furniture or bedding are required, thus increasing the cost and complexity of these technologies for effective BP monitoring. The following subsections highlight the principles and recent progress in these alternative methods.

#### 3.4.1. Ballistocardiography

**Figure 6:**
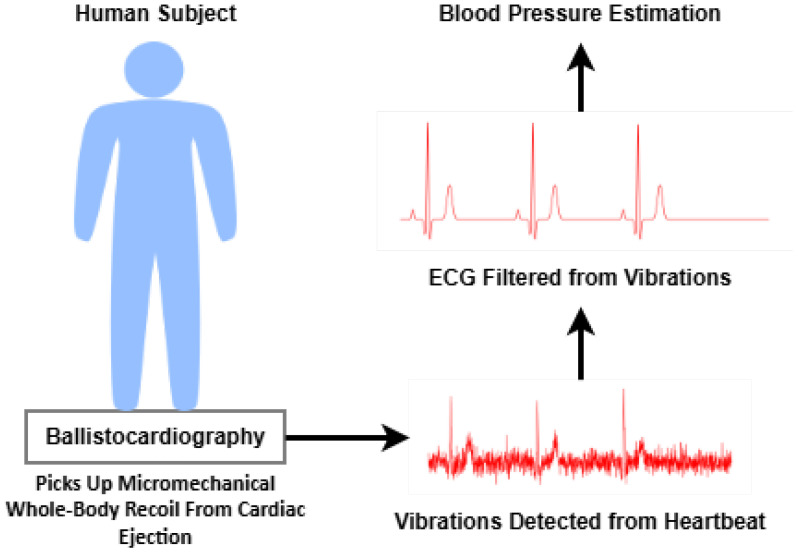
BCG method.

BCG is a technique that measures the body’s recoil forces generated by the cardiac ejection of blood into the vasculature. BCG signals can be recorded using bed-mounted, chair-integrated, or wearable sensors that detect minute displacements associated with cardiac cycles. These signals, which reflect the mechanical response of the body to cardiac activity, have been correlated with stroke volume and cardiac output, and indirectly with BP [49]. Recent developments have focused on using accelerometers and pressure sensors embedded in furniture or wearable systems to enable unobtrusive BP estimation [50]. However, the method remains sensitive to motion artifacts and requires signal averaging or advanced filtering techniques to ensure reliability. Figure 6 displays the BCG method.

#### 3.4.2. Seismocardiography

**Figure 7:**
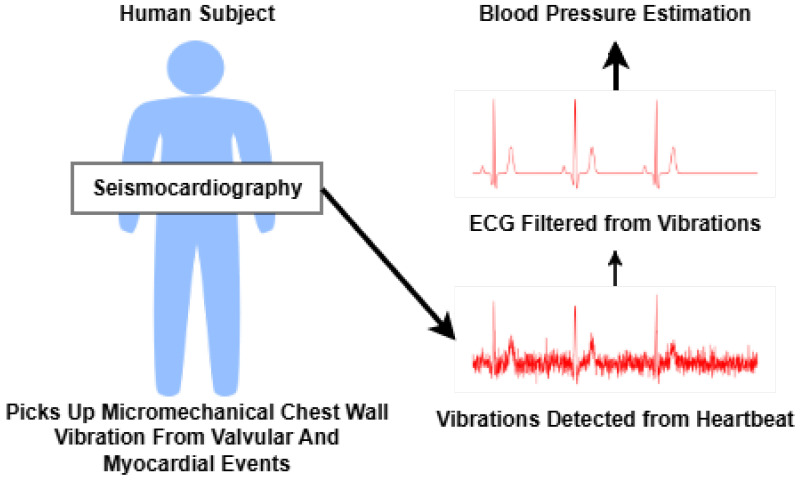
SCG method.

SCG refers to the measurement of chest wall vibrations resulting from cardiac mechanical activity, typically captured via accelerometers placed on the sternum. SCG captures the timing and amplitude of cardiac events, such as aortic valve opening and closing, which are influenced by hemodynamic factors, including BP. By analyzing portal intervals such as the PEP and Left Ventricular Ejection Time (LVET), SCG-based systems can infer BP variations [51]. SCG offers high temporal resolution and is increasingly adopted in wearable form factors; however, signal interpretation is complex due to inter-subject variability and the influence of respiration and posture. Figure 7 displays the SCG-based method.

#### 3.4.3. Capacitive and Electric Field Sensing

**Figure 8:**
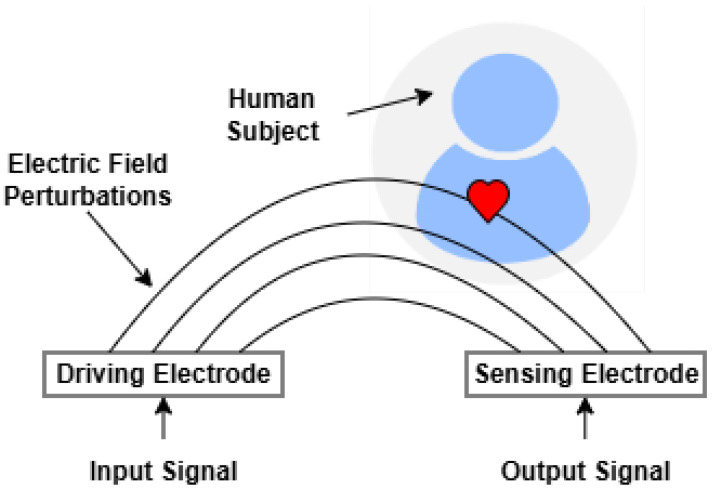
Capacitive and electric field sensing.

Capacitive and electric field sensing approaches measure changes in dielectric properties and displacement currents induced by cardiovascular motion. These sensors detect variations in body capacitance or electric field perturbations caused by arterial pulsations, chest expansion, or fluid shifts. Unlike traditional contact electrodes, capacitive sensors can operate through clothing or bedding, making them suitable for integration into ambient healthcare systems. Their application in BP monitoring typically involves feature extraction from displacement waveforms or integration with multimodal data streams [52]. Although promising in terms of comfort and deployment flexibility, these systems face challenges related to environmental noise and motion sensitivity. Figure 8 displays the capacitive and electric field sensing methods.

### 3.5. Multimodal Sensor Fusion for BP Estimation

Sensor fusion combines complementary modalities (e.g., radar and optical) to mitigate individual weaknesses of individual modalities [53]. Radar’s robustness to lighting and skin tone can offset rPPG’s sensitivity to illumination, while rPPG provides rich hemodynamic features absent in radar. Recent studies have demonstrated fusion strategies such as combining radar-derived chest ballistographic signals with camera-based PPG to compute PTT for BP estimation [54]. Sensor fusion can help with improving accuracy and resilience to environmental variability. However, by combining different sensors, the complexity of systems further increases. Synchronization needs to be considered so that data received from each individual sensor (to ensure that sensor data is synchronized for accurate readings) can be taken. Multiple sensors also increase the costs of systems as more sensors will need to be purchased. For real-time systems, low latency algorithms need to be implemented to reduce any delays in receiving data from each individual sensor. Any future research in the field of sensor fusion for effective BP monitoring should explore the implementation of high-signal-quality sensors and edge computing for real-time, low-latency fusion as well as for being validated against cuffless BP standards. Sensor fusion systems should consider any realistic accuracy gains against the cost and complexity of adding more sensors.

## 4. Measurement Principles for Cuffless BP Estimation

### 4.1. Pulse Wave Velocity

**Figure 9:**
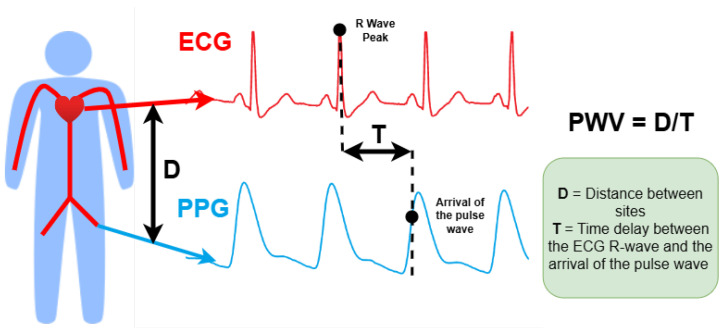
ECG and a PPG wave to calculate PWV.

PWV is the velocity at which a pulse pressure wave travels between arterial sites. It is defined as the distance between the femoral and carotid sites, divided by the arterial pulse wave delay (T). Figure 9 shows how the PWV is calculated using an Electrocardiogram (ECG) and a PPG wave. PWV and pulse wave delay are of interest in cuffless BP measurements, as BP can be estimated from them using a variety of physiological and regression-based models. A commonly used physiological model is the Moens–Korteweg formula: $PWV=\sqrt{Eh/\rho D}$, where *E* is the Young’s modulus of the arterial wall, *h* is the thickness of the arterial wall, ρ is the density of the blood, and *D* is the internal diameter of the artery. A more sophisticated physiology-based model is presented in [14]. Regression techniques provide an alternative method to extract BP from PTT using empirical equations such as $BP=k_{1}ln\left(PTT\right)+k_{2}$ and $BP=k_{1}^{\prime }/{\left(PTT-k_{2}^{\prime }\right)}^{2}+k_{3}^{\prime }$. However, methods of calculating BP from PTT that are based on physiological models (e.g., the Moens–Korteweg formula) have been shown to be more accurate than methods based solely on regression [55]. A key limitation of using PWV-based methods to measure BP, especially if physiological models are used, is the requirement of subject-specific calibration [32].

The first non-contact measurement of PWV was made by [56]. Here, PWV was calculated from the PTT between the heart and calf, using two radars centered at 6.05 and 5.63 GHz, respectively. Two frequencies were used to prevent interference between the radars. In [57], motions of the aortic artery were measured using the Doppler effect and a CW radar to calculate PTT.

Further work on BP measurement using a dual-radar setup is presented in [32,57]. Here, BP calculations were based on the PTT between the chest and wrist. The work in [32] achieved mean estimation errors for SBP and DBP of 0.87 ± 6.12 mmHg and 0.59 ± 3.78 mmHg, respectively, using a 77 GHz FMCW radar. The 2.45 GHz CW radars used in [57] directed at the chest and wrist were range-correlated and self-injection-locked, respectively. This was to optimally capture the different amplitudes of the vibrations at the different physiological sites. In both techniques, the vascular motion was determined from phase information, extracted from the demodulation of the received IQ signals.

A simplified experimental setup, which only required a single 77 GHz radar, was demonstrated in [58]. Here, BP was calculated from the Central Artery Pulse Transit Time (caPTT) between the chest and neck. An additional advantage of this technique is the increased accuracy of BP measurements based on PTT between the aorta and carotid artery over using PTT between the aorta and peripheral arteries (e.g., wrist or fingers). The mean estimation errors for SBP and DBP were 5.54 ± 7.62 mmHg and 4.68 ± 6.15 mmHg, respectively.

Camera-based techniques have also been explored as a means of cuffless PTT measurement for BP estimation. A brief overview of the key advances made in cuffless BP estimation between 2016 and 2021 is given in [59]. This paper reviewed six camera-based approaches that were based on obtaining the PPT from PPG. One study analyzed achieved estimation errors for SBP and DBP of 0.01 ± 7.71 mmHg and 0.18 ± 5.54 mmHg, respectively. However, there were only five subjects in this data set.

### 4.2. Micro-Doppler Effect in Radar Systems

**Figure 10:**
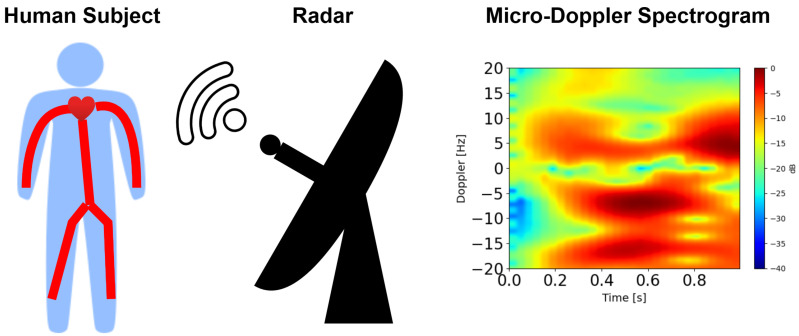
Spectrogram produced from the micro-motions of a human target using radar.

The micro-Doppler effect refers to frequency modulations, due to micro-motions of a target, which are superimposed onto the Doppler frequency shift due to the target’s translation. The micro-motions result in sidebands around the Doppler frequency, which can be analyzed using time-frequency analysis. Due to the time-varying nature of micro-Doppler signals, FFT is an inadequate tool for their analysis [60]. Consequently, the STFT is a commonly used method of producing spectrograms from micro-Doppler signals. Figure 10 shows a spectrogram produced from the micro-motions of a human target.

The micro-Doppler effect has been studied for numerous healthcare applications such as activity classification and breathing rate measurements. Studies such as [61,62] employed a 2.46 GHz and a 24 GHz radar, respectively, for activity classification using the micro-Doppler effect. These studies achieved accuracies of 79.2% and 93.1%, respectively. Further work on activity classification using a 24 GHz radar and the micro-Doppler effect found that breathing patterns can be discerned from the displacement vs. time graph for a stationary test subject, potentially allowing breathing rate measurements to be made [63]. The micro-Doppler effect has also been used to measure breathing rates with an estimation accuracy of 93.75% [64].

The feasibility of using the micro-Doppler effect for cuffless BP measurements was demonstrated in [42]. Here, a 300 GHz, CW radar was selected to improve sensitivity to minute chest motions. This approach achieved mean relative errors on estimates of heart rate, SBP, and DBP as low as 4.57%, 8.04% and 8.3%, respectively, in comparison to ground-truth measurements. A notable innovation of this work is the calculation of BP from mechanical heart sounds, which are extracted using a discrete wavelet transform. Of the ML techniques tested, Support Vector Machine (SVM) and bagging performed the best for SBP and DBP, respectively, especially when they were trained on frequency-domain features. While this work demonstrates that the micro-Doppler effect is a promising research avenue for cuffless BP measurement, it suffers from three limitations. Only eight test subjects were studied. Furthermore, test subjects were required to hold their breath to reduce noise, and low and high BP values were underestimated and overestimated, respectively.

### 4.3. Ballistographic and Hemodynamic Responses

BCG is the measurement of the ballistic force of the human body generated by heartbeats. Research has shown that BCG can be observed using camera technology in a cuffless manner. These measurements can be used to estimate BP [65]. Kim, Chang-Sei, et al. [66] made use of force plates for BCG measurements of the SBP and DBP readings for 22 healthy volunteers. The timing and amplitude of BCG signals are able to provide diastolic and pulse pressure. Adding these values together gives the SBP. The combined BCG features achieved an RMSE within acceptable limits for clinical applications, indicating the potential for accurate BP monitoring. The limitations of this paper are that there were only readings taken from healthy subjects, so data lacked hypertension and hypotension samples. Additionally, subjects would need to stand on the force plate, making the proposed system impractical with individuals with mobility issues. Hemodynamic responses are how the body adjusts blood flow in response to activity for the delivery of nutrients to muscle tissue. Nutrients such as oxygen [67] and glucose [68] are needed by tissues strained during activities. Hemodynamic data can be used to infer BP as it represents the dynamics of blood flow including cardiac output, stroke volume, and vascular resistance, all of which influence BP. The work of [69] made use of smartphone PPG sensors to detect normalized blood volume and pulse rate to estimate BP. Results showed correlation coefficients of 0.934 for mean BP between traditional cuff-based methods and estimated BP.

## 5. Signal Processing and AI-Based Estimation Techniques

### 5.1. Time-Domain Analysis

Time-domain analysis involves examining how signal amplitude varies over time. This approach is widely used across sensing modalities such as radar, PPG, ECG, and BCG to extract features relevant to BP estimation. Common features include PAT, pulse width, rise time, foot-to-peak time, and area under the curve [70,71,72]. For instance, Wang, Ruiping, et al. [73] utilized ECG and PPG signals from the MIMIC database [74] to derive PAT and heart rate for BP prediction.

In addition to raw PPG signals, their derivatives (first to fourth order) are widely used in BP estimation and vascular health assessment. Derivatives are calculated by differentiating the signal. The raw PPG signal shows blood volume changes over time. The first derivative measures how fast the signal is changing at each point. The second derivative measures how the slope itself changes (curvature), which highlights inflection points in the waveform. Higher derivatives (third, fourth) capture even finer details of the waveform’s shape [75].

However, time-domain signals are susceptible to noise from sensor limitations, motion artifacts, and respiration. Signal denoising is essential to ensure accurate feature extraction. Radar-based studies have applied Principal Component Analysis (PCA) to isolate heartbeat signals from environmental noise [76,77]. Kalman filtering and bandpass filtering have also been used to enhance radar signal quality [78,79]. Wavelet transforms, which detect localized patterns in time-series data, are effective in separating heartbeats from random motion artifacts [80].

In optical methods such as rPPG, time-domain signals are derived from video frames capturing skin color changes due to blood flow. The green channel is often preferred due to its optimal absorption characteristics [81,82]. Multivariate techniques like Chrominance-based rPPG (CHROM), PCA, and Independent Component Analysis (ICA) help to isolate PPG signals from noise and lighting variations [83,84]. These methods have demonstrated robustness in extracting physiological signals from facial videos under varying conditions.

### 5.2. Frequency-Domain Analysis

Frequency-domain analysis focuses on identifying frequency components within signals, typically using FFT [85]. This transformation enables the extraction of features such as Power Spectral Density (PSD), which quantifies signal energy across frequencies. PSD has been used to estimate BP from PPG signals captured via smartphones [86] and radar signals processed to isolate heart rate frequencies [87,88].

The spectral analysis of BP-related oscillations provides insight into autonomic regulation. Cardiac oscillations are categorized into Very Low-Frequency (VLF, <0.04 Hz), Low-Frequency (LF, 0.04–0.15 Hz), and High-Frequency (HF, 0.15–0.4 Hz) bands [89,90]. LF oscillations are associated with sympathetic nervous activity, while HF oscillations reflect parasympathetic and respiratory influences [91,92]. The sympathetic nervous system elevates BP during stress or exertion, whereas parasympathetic activity promotes relaxation and BP reduction [93].

Heart Rate Variability (HRV), derived from R–R intervals in ECG or PPG signals, reflects the balance between these autonomic branches. High HRV is indicative of cardiovascular health, while low HRV may signal hypertension or stress. Radar-based systems have successfully estimated HRV in real time, even in dynamic environments such as vehicle cabins [94,95].

### 5.3. AI-Based Approaches

AI has become central to BP estimation, enabling models to learn complex relationships between physiological signals and BP values. AI techniques are typically applied to features extracted from time-domain and frequency-domain analyses, or directly to raw signals.

#### 5.3.1. Deep Learning Models

DL models such as Convolutional Neural Networks (CNNs) and Long Short-Term Memory (LSTM) networks have shown strong performance in BP estimation tasks. CNNs are effective in capturing spatial patterns in PPG waveforms, while LSTMs model temporal dependencies. Hybrid CNN–LSTM architectures have been applied to the MIMIC-III dataset [74], achieving high accuracy in BP prediction [96,97,98]. Sky Labs recently published a deep learning-based system (PPG2BP-net), a novel CNN that utilized PPG data from over 4000 subjects, achieving accurate continuous BP tracking with arterial ground truth (SBP error 1 mmHg; DBP error 0.5 mmHg) [99].

Radar-based studies have also adopted DP. Ran, You, et al. [100] and Han-Trong, Thanh, and Hoang Nguyen Viet [87] used CNN and LSTM models to estimate BP from radar signals. Wang, Pengfei, et al. [23] implemented a CNN–Bidirectional LSTM hybrid on 24 GHz radar data, improving BP estimation accuracy. Bidirectional LSTM models have been used to detect HRV from radar signals, demonstrating robustness in dynamic environments [101].

In the domain of rPPG, CNN-based models have been used to estimate heart rate and BP from RGB video data [102,103]. These models leverage spatial and temporal features from facial videos, with CHROM and ICA preprocessing enhancing signal quality.

#### 5.3.2. Traditional Machine Learning

Traditional ML methods such as SVM, K-Nearest Neighbors (KNN), and Random Forests have also been applied. Zhang, Yue, and Zhimeng Feng [104] trained an SVM on PPG data, outperforming linear regression and neural networks in continuous BP monitoring. Radar-based HRV estimation has benefited from ML models trained on LF and HF oscillations [105].

Camera-based systems have used ML to estimate BP from PTT and rPPG features. Kawasaki, Sae, et al. [106] combined visible and near-infrared (NIR) imaging with support vector regression to predict SBP and DBP, achieving improved accuracy.

#### 5.3.3. Feature Extraction and Model Evaluation

AI models are not only used for prediction but also for feature extraction. CNNs and LSTMs have been employed to isolate clean PPG signals from noisy data. Tian, Zhonghe, et al. [107] used a CNN to extract PPG features and estimate mean BP with a MAE of 2.03 mmHg, meeting grade A standards under the BHS protocol. The model was trained on the MIMIC-III dataset, which includes ECG and PPG data from more than 50,000 ICU patients.

LSTM models have also been used to detect clean pulse waves from radar signals distorted by respiration [108]. These models enhance signal quality and improve BP estimation accuracy in non-contact settings.

#### 5.3.4. Challenges and Future Directions

Despite promising results, AI-based BP estimation faces several challenges:

- Dataset limitations: Many models rely on clinical datasets that may not generalize to real-world conditions. Public datasets like MIMIC-III are valuable but lack diversity in sensor types and environments.
- Computational complexity: DL models require significant computational resources, limiting their deployment on wearable or edge devices.
- Model interpretability: Black-box models hinder clinical adoption due to lack of transparency in decision-making.
- Performance variability: Accuracy can vary across populations, sensor modalities, and environmental conditions.

Future research should focus on developing lightweight, interpretable models trained on diverse, multimodal datasets. Benchmarking studies with standardized evaluation metrics (e.g., MAE, RMSE, correlation coefficients) are needed to compare model performance across modalities. Additionally, real-time feasibility and energy efficiency should be considered for deployment in wearable and remote monitoring systems.

## 6. Challenges

### 6.1. Physiological and Individual Variability in BP Estimation

Several papers have investigated the impacts of physiological and individual variability on BP estimation. For a device to be practical, it must be known how these influential factors impact the estimation accuracy. This also informs whether subject-specific calibration would realistically be required or not for the device to be robust against different users.

Fang et al. attempted to develop a practical BP estimation system via rPPG and ML by incorporating a dataset with diverse ethnicities (African, Asian, European), skin tones (all six Fitzpatrick scale categories), ages (8 to 91), environments (both indoor and outdoor), and BPs [109]. Particularly for rPPG, as it is a camera-based technology, testing on different skin tones and environments is crucial. Additionally, they displayed the distribution of these factors in their dataset for clarity. The study found that accuracy was lower on test data of high and low BPs, due to the comparative lack of these BPs in the training data. This emphasizes the importance of having enough training data for high and low BPs. In addition, they found that accuracy was lower when estimating the BP of those of African descent, concluding that darker skin tones may have an impact on extracting effective rPPG signals. This is a significant factor to consider when developing a device, as it must be accurate to all skin tones, an issue that is unique to camera-based monitoring. The study also found that age was not a significant factor in the accuracy achieved for BP estimations.

There is also uncertainty about the impact of cardiac arrhythmia on the accuracy of BP estimation, as there is a lack of papers using data from subjects with cardiovascular disease. This was addressed in [110] by collecting rPPG data on subjects with cardiovascular disease to understand the effects of physiological differences. They collected data on participants with a wide range of BP values, as limiting data to only normotensive BPs would constrain the validity of the model and could lead to the model predicting mean values. In addition, they aimed to collect data of not just young and healthy participants, but also those who are older and those with cardiovascular disease. Cardiovascular disease and age will impact factors like arterial stiffness, which would have an effect on the waveform of the data collected. The impact of cardiovascular disease-related medications was also highlighted in [110]. These medications can cause reduced cardiac contractility and decreased systemic vascular resistance, which may affect the signals obtained. In addition, abnormal heart rhythms like atrial fibrillation will also impact the pulse pressure and hence the waveform obtained.

To investigate this, Curran, Theodore, et al. [110] recorded ECG alongside rPPG in order to observe differences in heart rhythm and the subsequent effects on BP estimation. They used two models for estimation: a hybrid model and a PPG-only model, with the former including demographic features (such as ethnicity, age, and medical history) for calibration. This is useful for comparing and investigating the need for demographic calibration in rPPG systems. Having data from 143 participants, this study is also higher in subject count than most, which also helps in understanding the generalizability of the model. Interestingly, they found that demographic calibration did not significantly increase BP estimation accuracy for the algorithm they had implemented. In addition, they did not observe any differences between clean and noisy training, where the latter involves using data from all participants. This result is important as it means that the physiological differences did not cause any significant challenges in estimation accuracy. Finally, they also found that cardiac arrhythmia did not significantly affect the accuracy of BP estimations either. However, the sample sizes for those with arrhythmia were much smaller than for those with normal sinus rhythm (<30 vs. >180), which greatly affected the interpretability of the results. There are additional limitations; there was no truly healthy cohort to analyze as the control group, as all subjects either had cardiovascular disease or were at risk of it. In addition, the MAEs were above 11 mmHg for all models, meaning there is a broad margin of error. Perhaps in a system that estimates with more accuracy, these differences among populations would be more significant. Overall, however, this study is an important step in the direction of proper validation for cuffless BP estimation, ensuring robustness across various populations.

In terms of radar use, there has been less research into testing on participants with cardiovascular disease or varying physiology. However, some studies do investigate some physiological differences, such as breathing patterns. Movement due to respiration is known to bring noise to radar data; hence, researchers must test whether systems are robust against different noise brought by different breathing patterns. This was investigated in [44,111] by using data from participants in various physiological states such as the Valsalva manoeuvre and apnea. Both achieved successful results regardless of these states, due to the robust signal processing employed. Importantly, [112] collected radar data from the wrists of a diverse set of participants, considering BMI, age, skin tone, and gender. They were able to obtain accurate BP predictions regardless of these differences in physiology. BMI bias did not affect the accuracy, which showed that, for their system, the fat layer was not significantly absorbing the radar waves. However, they found a decrease in accuracy for males, stating that this may be due to the higher absorption rates of males’ thicker skin. They found the same for younger participants, which they associated with absorption from increased hydration levels. Finally, they found that there was less accuracy for darker skin tones. However, this result is difficult to interpret as there were only four subjects with darker skin tones. In future work, it may be useful to have a high number of participants for each physiology type, in order to better validate the correlation to estimation accuracy. An area that is missing for radar-based systems is an investigation into the impacts of cardiovascular disease. However, studies like [112] have shown robustness across various BP ranges.

### 6.2. Motion Artifacts and Environmental Interference

All studies discuss the challenges of noise from both motion and the environment. In the case of radar sensing, motion is a major challenge as it corrupts the signal being targeted. For cameras, both motion and lighting conditions are extremely influential in determining the accuracy of estimations made, as they can hide important details in the signal.

For radar-based systems, researchers have developed various methods to address the challenge of noise and motion artifacts. Studies initially use filtering such as lowpass, bandpass, or discrete wavelet transform in order to target cardiac activity [42]. Some also opt for an FMCW or UWB radar for range resolution in order to filter out noise from outside the target range [32,36]. Furthermore, some studies have used further signal-processing steps to better eliminate noise. A peak detection algorithm, to detect aliasing, was adopted by [36]. A time-varying filter-based empirical mode decomposition was used by [43] to decompose the signal into several intrinsic mode functions, consequently minimizing noise by extracting purely the modes correlated to the target pulse wave. Zhongrui Bai et al. [41] selected data by developing a signal quality index, using a harmonic summation algorithm, which allowed the automatic optimization of the dataset by discarding recordings with too low of an SNR. This determined which data would be used for training and testing the ML model. While this advanced algorithm yielded very strong results, it may have limited practical usability for a real-time device, as it showed that, for ideal accuracy, only around 75% of data could be used, which may be limiting when attempting to obtain real-time measurements. To obtain optimal results, ref. [113] also discarded noisy data by conducting morphology selection. This may be more suitable for real-time use, as only single-pulse waves are removed from recordings containing many consecutive pulse waves. However, an issue is that this study involved much longer recordings, on the scale of minutes, in order to obtain enough useful data. In addition, it was not stated what percentage of data was retained after this process. The work in [114] used adaptive filtering for each recording to first target the frequency of the wrist pulse via FFT, before designing a finite impulse response filter. This yielded better results than using a fixed cutoff frequency. An example of another advanced filtering technique is [32], which uses a CNN-based translation filter, which learns the filter’s ideal coefficients in order to recover the fine-grained arterial pulse signal from noisy phase data. By doing this, they showed that they were able to recover the ground truth arterial pulse signal from the radar signal, with much higher similarity than when using a simple finite impulse response filter.

To evaluate their BP estimation system, ref. [45] recorded data in multiple environments and left one environment out of the training set completely, in order to use it only for the test set. Through this, they were able to show that their system worked in a new environment, on which the ML model had not been trained. In addition, they tested different radar placements. For their system, they found that radar angle can be acceptably flexible up to 45°. They also found that increased distance from the subject decreases accuracy, while finding an appropriate maximum range of use of 90 cm for acceptable accuracy. This type of evaluation is extremely valuable as it ensures that the ML model is tested against any overfitting of the training data and that the estimation system can be used in various environments and setups. These factors are incredibly important in ensuring the practical use of the device being developed. To further enhance evaluation, testing can be carried out in various real-world clinical environments where the device may be used. Yong Wang et al. [43] also evaluated different distances and angles, finding that distance must be kept under 15 cm and angle under ±30° for their system to meet the AAMI standards.

Aside from radar, rPPG is a signal that can also experience noise due to motion as well as interference due to light. As it is a light-based sensing modality, the lighting conditions must be appropriate to obtain cuffless BP predictions. Low-light conditions will obscure the details that the rPPG is targeting [106,115]. This may be limiting in clinics and at home, as these would require these devices to only be used in specific environments.

To combat the impact of lighting, ref. [106] used an RGB–NIR camera rather than just RGB. Including NIR allows for better robustness and accuracy under diverse lighting conditions. However, the necessity of NIR contrasts with rPPG’s advantage of being software-only and usable in commercial cameras and smartphones. In addition, these cameras are much more expensive than most radars used in other studies, which do not have any influence from light. Ismoil Odinaev et al. [115] successfully showed that adjusting camera settings can allow for better mitigation of the impact of low-light conditions and improved accuracy in vital sign estimation. A DL-based image enhancement model, inspired by the Retinex theory, was used by [116] to combat the effect of lighting conditions in an automated manner. This advanced algorithm allowed for accurate prediction of vital signs. However, this involvement of an extra convoluted step of processing may be unnecessary when other sensing modalities like radar can be used. In addition, these algorithms have not been conducted yet in the case of BP estimation. Motion artifacts are also an influence on the use of rPPG signals, with most studies using at least a bandpass filter [107,117]. However, some studies use further processing. To get rid of recorded cardiac cycles corrupted by motion artifacts, ref. [106] calculated the mean total duration and mean amplitude of all cardiac cycles, and then excluded any cardiac cycles which were outside of one SD difference. In this case, the recordings were only 20 s each, indicating that this time of recording was long enough to obtain enough usable cardiac cycles per recording. Zhonghe Tian et al. [107] also used a similar signal quality evaluation by eliminating signals with excessive amplitude and saturation. However, neither study stated what percentage of data was retained after this process.

Overall, motion and environmental interference is a major challenge in BP estimation. Solutions for minimizing these effects range from advanced signal processing, e.g., DL-based filtering [41], to differences in the practical setup, e.g., the use of an RGB–NIR camera or an FMCW radar [79,106]. An advantage of radar in this context is that the challenge of lighting conditions does not exist. They can be used in any environment, no matter the lighting. However, regardless of this, rPPG detection has great capabilities for integration into commercial cameras and self-use with appropriate lighting. In addition, its capability to be software-only can be advantageous for pricing. On the other hand, for practical prevalent use in clinics, there is a necessity for robust and reliable use in diverse scenarios, in which case radar may be more appropriate. Radar would also be more suitable if reliably consistent use is needed in self-use cases as well.

### 6.3. Regulatory and Clinical Adoption Challenges

For clinical adoption and regulatory compliance, the validation of different environments and individual variability is crucial. Research on this has been overviewed in the previous sections. Aside from this, it is relevant to understand the possible challenges in the clinical adoption of such devices.

One challenge to investigate is practical use. Depending on how the device must be used, there would be many questions on how that would apply in a clinical setting. For instance, a camera-based rPPG device may have limitations in lighting, thus limiting wide deployability. On the other hand, a radar-based chest recording device may have limitations of distance, angle, and foreign interference, making the requirements for accurate measurement too stringent. In both cases, the literature has recognized these issues and hence investigated ways to improve these aspects of the technology [45,106]. When validating a device for clinical use, these types of limitations would have to be thoroughly investigated to understand the practicality of the device. Especially in self-use for patients, recording needs to be easy and not overly specific, as the idea is to introduce a more convenient method of recording BP rather than introducing new challenges. Ideally, a device would be able to, for example, be used in home lighting conditions or should have a fair amount of flexibility in terms of angle and distance for recording. In addition, if a device needs to record a specific location, such as neck, wrist, or chest, there would need to be a way to easily set up the device to target those locations. Another important factor is cost. Some devices may be much more expensive than a cuff-based device, such as those including radars. There needs to be a reason to spend more on these devices, emphasizing the importance of comfort and convenience through practicality. The importance of this is further emphasized when some users may be skeptical of new technology.

Some studies also emphasize the importance of the explainability of the devices developed. For example, ref. [45] emphasizes that, in decision-sensitive medical scenarios, there needs to be a sound physiological basis for the readings obtained. They argue against the use of black-box models, which use DL to train models in a “brute force” manner. Hence, they use a feature that uses the compliance of the arm arteries, arguing that this has a clearer physiological basis.

Various guidelines exist to classify the devices developed. Many studies test against the BHS or AAMI guidelines, which were overviewed in Section 2 [118]. The ESH recommendations are also very important as they are targeted specifically to cuffless devices [27]. This means that they emphasize conducting various tests such as position tests, post-exercise tests, and post-treatment tests. They also encourage other specific tests for devices that require calibration. While the ESH guidelines recommend these tests primarily for wearable cuffless devices, such tests are encouraged in order to fully validate any BP monitoring device developed.

## 7. Discussions

The evolution of cuffless BP monitoring has brought forward a diverse set of technologies, each with unique strengths and limitations, as shown in Table 4. A comparative evaluation of key sensing modalities reveals meaningful contrasts. rPPG, for instance, offers a low-cost, camera-based solution with the potential for wide deployment via smartphones. However, it faces challenges related to lighting variability, motion artifacts, and skin tone bias, raising questions about accuracy and fairness. Radar-based systems, while more robust to ambient lighting and privacy-respecting in their passive nature, introduce higher hardware complexity and cost. Acoustic and ultrasound methods offer additional opportunities but remain constrained by environmental noise, shallow penetration depth, and integration challenges in real-world settings.

Calibration remains a significant barrier to the clinical adoption of cuffless BP technologies. The performance of many existing models degrades across demographic and physiological variations, necessitating population-specific tuning. This limits scalability and undermines trust. There is an urgent need for calibration-free or calibration-minimized approaches that can generalize across diverse populations. This focus on developing calibration-free systems has been seen in recent research, with success across various sensing modalities. Techniques such as federated learning may also offer a path forward by enabling distributed model adaptation without centralized data pooling, yet they still implicitly introduce forms of calibration and require further exploration.

Clinical validation also remains underdeveloped. Most studies suffer from small sample sizes, homogeneous populations, or limited diversity in hypertensive status. The lack of standardized evaluation protocols and the difficulty of comparing continuous, real-time estimation methods against intermittent gold-standard cuff-based measurements make clinical benchmarking complex. For true integration into healthcare workflows, future studies must employ rigorous, large-scale validation with diverse cohorts and clinically relevant endpoints.

On the regulatory and ethical front, cuffless methods raise novel concerns. Visual data collection via rPPG, even when anonymized, may be perceived as intrusive by patients. Similarly, explaining the use of radar or RF-based technologies to laypersons may provoke skepticism or unease, especially among individuals with concerns about electromagnetic exposure. Trust in these technologies will depend not only on technical performance but also on clear communication, robust data governance, and thoughtful design that minimizes perceived invasiveness.

Despite these challenges, hybrid systems that fuse multiple sensing modalities (e.g., combining radar with rPPG or inertial sensors) offer a promising route toward robustness. Such systems can mitigate the weaknesses of individual modalities and adapt to complex environments. However, they also increase computational load, system complexity, and ethical considerations due to multimodal data collection. Careful system design, sensor synchronization, and cost–benefit analysis are crucial for hybrid approaches to succeed.

AI, particularly DL, has emerged as a central enabler of cuffless BP estimation. While high-performance results are achievable, these models often lack transparency and interpretability—qualities essential for clinician acceptance. By contrast, signal processing techniques grounded in physiological models are more explainable but less flexible. A synergistic integration of physiological insight with data-driven learning may yield systems that are both accurate and trustworthy.

Deployment in real-world settings introduces further complexity. Devices must maintain performance across varying lighting conditions, motion artifacts, user postures, and levels of operator expertise. Practical considerations such as device alignment, user training, and maintenance are rarely addressed in the literature but are critical for successful clinical integration. Moreover, the question of accountability, who bears responsibility when a system fails, is paramount and remains underexplored.

Cuffless BP technologies offer compelling opportunities for improving access in underserved communities and low-resource settings. Smartphone-based solutions, in particular, could facilitate remote monitoring, community-level screening, and telemedicine. However, developers must prioritize algorithmic fairness, data inclusivity, and affordability to prevent reinforcing health disparities.

Looking forward, transitioning from proof-of-concept to clinical utility will require coordinated efforts across the research, clinical, and regulatory communities. Key priorities include the creation of open-access datasets, standardized benchmarks, and validation protocols; the design of intuitive, user-friendly devices; and the deployment of pilot studies in diverse, real-world contexts. Addressing these gaps will be essential for achieving the scalability, compliance, and long-term adoption of cuffless BP monitoring technologies.

In summary, based on the technologies reviewed, radar-based and rPPG systems emerge as the most plausible modalities for cuffless BP monitoring. Both have demonstrated strong accuracy in the current literature and offer distinct advantages for continuous, non-invasive measurement. Notably, these approaches show potential for calibration-free operation, which is critical for scalability and user convenience. Furthermore, ongoing advancements in AI and signal processing are expected to enhance the ability to extract robust physiological features from raw radar and optical signals, enabling the accurate estimation of SBP and DBP in real-world settings. Future work should prioritize the development of large, diverse, open-access datasets; the creation of generalizable models with minimal calibration requirements; and the establishment of standardized benchmarking and validation protocols aligned with clinical practice. Equally important will be the design of intuitive, user-friendly hardware interfaces and the development of ethical frameworks that foster transparency and trust. With coordinated research, clinical integration, and regulatory collaboration, cuffless BP monitoring can evolve into a scalable, compliant, and widely adopted solution for ambient cardiovascular health monitoring.

## 8. Conclusions

This paper has reviewed the current landscape of cuffless BP monitoring technologies, highlighting the wide spectrum of sensing modalities, from camera-based rPPG and RF radar systems to emerging acoustic and ultrasonic techniques. We have explored their respective advantages, limitations, and multifaceted challenges related to calibration, clinical validation, regulatory compliance, and real-world deployment. Emphasis has been placed on the role of AI and signal processing in enhancing estimation accuracy, and on hybrid approaches that combine complementary sensing modalities. We have also underscored the broader implications for global health equity and ethical deployment. While substantial progress has been made, cuffless BP monitoring technologies remain in a formative stage. For them to transition from experimental setups to clinical and consumer adoption, several pressing issues must be addressed.

## Figures and Tables

**Table 1:** Accuracy classification of BP devices based on mean difference and SD (mean ± SD).

Accuracy Level	Mean Difference (mmHg)	SD (mmHg)	Failure Chance
High Accuracy	0	±3–6	<14%
Moderate Accuracy	4	±5	<28%
	6–8	±5	
Low Accuracy	0	±10–12	<94%
	4–6	±8	

**Table 2:** Classification of accuracy based on absolute error range of readings.

Absolute Error Range	Accuracy Category
0–5 mmHg	Very Accurate/No Error of Clinical Relevance
6–10 mmHg	Accurate/Slightly Inaccurate Clinically
11–15 mmHg	Moderately Inaccurate
>15 mmHg	Very Inaccurate

**Table 3:** Grading of test devices based on absolute error agreement with a standard device.

	Grade			
Absolute Error Between Reference and Test Devices	A	B	C	D
≤5 mmHg	60%	50%	40%	<40%
≤10 mmHg	85%	75%	65%	<65%
≤15 mmHg	95%	90%	85%	<85%

**Table 4:** Sensing modality comparative analysis table.

Modality	Contactless?	Calibration-Free?	Cost & Complexity	Environmental Sensitivity	Clinical Readiness
Radar	Yes	High potential	Medium–High	Medium	Moderate
rPPG	Yes	Limited	Low	High	Moderate
Thermal Imaging	Yes	Limited	High	High	Low
Acoustic/Ultrasound	No	Limited	Medium	Medium	Moderate
BCG/SCG	No	Limited	Medium	High	Low
Capacitive/Electric Field	Yes	Limited	Medium	Medium	Low

## Data Availability

No new data were created or analyzed in this study. Data sharing is not applicable to this article.

## Abbreviations

The following abbreviations are used in this manuscript:

AICCET	Accelerating Impact of Community healthCarE in Tayside
AHA	American Heart Association
AI	Artificial Intelligence
AAMI	Association for the Advancement of Medical Instrumentation
BCG	Ballistocardiography
BP	Blood Pressure
BHS	British Hypertension Society
caPTT	Central Artery Pulse Transit Time
CHROM	Chrominance-based rPPG
CW	Continuous Wave
CNN	Convolutional Neural Networks
DL	Deep Learning
DBP	Diastolic BP
ECG	Electrocardiogram
EPSRC	Engineering and Physical Sciences Research Council
ESH	European Society of Hypertension
FFT	Fast Fourier Transform
FMCW	Frequency-Modulated Continuous Wave
HRV	Heart Rate Variability
HF	High-Frequency
ICA	Independent Component Analysis
ISO	International Organization for Standardization
KNN	K-Nearest Neighbors
LVET	Left Ventricular Ejection Time
LoA	Limits of Agreement
lstm	Long Short-Term Memory
LF	Low-Frequency
ML	Machine Learning
MAE	Mean Absolute Error
NIR	Near-Infrared
PPG	Photoplethysmography
PSD	Power Spectral Density
PEP	Pre-Ejection Period
PCA	Principal Component Analysis
PAT	Pulse Arrival Time
PTT	Pulse Transit Time
PWA	Pulse Wave Analysis
PWV	Pulse Wave Velocity
RGB	Red, Green, and Blue
rPPG	Remote PPG
RMSE	Root Mean Squared Error
SCG	Seismocardiography
SD	Standard Deviation
SVM	Support Vector Machine
SBP	Systolic BP
UWB	Ultra-Wide Band
VLF	Very Low-Frequency
